# Clinical Utility of Stillbirth Investigations in Australia: A Cohort Study

**DOI:** 10.1111/ajo.70144

**Published:** 2026-05-13

**Authors:** Tania Marsden, Mu Cheng, T. Yee Khong, Jane E. Dahlstrom, David Ellwood, Ali Moghimi, Stacey Prystupa, Cecilia Obrien, Fathima S. Cassim, Skye Martin, Michael Coory, Frances M. Boyle, Heidi Shukralla, Alison L. Kent, Ashwati Krishnan Varikara, Patricia Hannaford, Joanne Frost, Joanna Perry‐Keene, Gretchen Pomare, Yin Ping Wong, Geok Chin Tan, Vicki Flenady, Jessica Sexton

**Affiliations:** ^1^ NHMRC Centre of Research Excellence in Stillbirth, Mater Research Institute University of Queensland Brisbane Australia; ^2^ SA Pathology Women's and Children's Hospital Adelaide Australia; ^3^ ACT Pathology, Canberra Health Services and School of Medicine and Psychology ANU College of Health and Medicine Canberra Australia; ^4^ Department of Histopathology The Children's Hospital Westmead New South Wales Australia; ^5^ Clinical School, the Children's Hospital at Westmead, Faculty of Medicine and Health University of Sydney Westmead New South Wales Australia; ^6^ BUPA Melbourne Australia; ^7^ Consultant Maternal Fetal Medicine Specialist Townsville University Hospital Townsville Queensland Australia; ^8^ ATSICHS Brisbane Australia; ^9^ Registrar Obstetrics and Gynaecology Townsville University Hospital Townsville Queensland Australia; ^10^ Public Health Registrar, Linear Clinical Research Perth Western Australia Australia; ^11^ Pediatrics University of Rochester School of Medicine and Dentistry Australian National University Canberra Australia; ^12^ NSW Health Pathology John Hunter Hospital Newcastle New South Wales Australia; ^13^ Anatomical Pathology Capital Pathology. Canberra Australia; ^14^ Obstetrics and Gynaecology Registrar and Doctoral Candidate, Women's and Newborn Services, Royal Brisbane and Women's Hospital, Faculty of Medicine The University of Queensland Brisbane Australia; ^15^ Pathology Queensland Brisbane Queensland Australia; ^16^ Te Whatu Ora, Health New Zealand Auckland New Zealand; ^17^ Department of Pathology, Faculty of Medicine Universiti Kebangsaan Malaysia Kuala Lumpur Malaysia; ^18^ Deputy Dean of Research and Innovation of the Faculty of Medicine Universiti Kebangsaan Malaysia Kuala Lumpur Malaysia

**Keywords:** autopsy, death classification, placenta, stillbirth

## Abstract

**Background:**

Stillbirths impact over two million parents globally every year. Despite current knowledge, technology, and investigations, many stillbirths remain unexplained and are not fully investigated. An important step forward in addressing this gap is determining which investigations produce the highest utility in identifying the cause of death (COD).

**Aims:**

The objective of this study is to identify and define the usefulness or utility of investigations in determining COD among a cohort of stillbirths.

**Materials and Methods:**

Prospective cohort study from 2013 to 2018 comprising 695 stillbirths. An expert panel blinded to COD assessed the clinical utility of investigations using a purpose‐built tool. A stepwise approach was applied, categorising investigations into three groups: clinical and laboratory investigations; placental pathology; and autopsy examination. Clinical utility of the investigations was defined as the proportion of cases in which each test contributed to the identification of the COD.

**Results:**

Placental pathology had the highest clinical utility (87%), followed by comprehensive maternal history (82%), genetic analysis (75%), maternal blood investigations for infection (64%), fetal‐maternal haemorrhage (FMH) (57%), and fetal autopsy (47%). Placental pathology and genetic analysis were useful across all clinical scenarios, while autopsy was most beneficial when the clinical scenario was unknown. A COD was established in 528 cases (76%), with 310 cases (47%) identified post‐placental examination.

**Conclusions:**

The investigations with the highest clinical utility were placental pathology, comprehensive maternal history, genetic analysis, maternal blood investigations, FMH, and fetal autopsy. This study supports a core set of investigations, supplemented by selective tests based on the clinical scenario.

## 1 Introduction

Globally, it is estimated that 2.6 million stillbirths occur annually, with 98% occurring in low‐ and middle‐income countries (LMIC), with approximately half occurring in the intrapartum period [1]. In high income countries (HIC), most stillbirths are born during the antepartum period and many of these deaths are potentially preventable [1]. In Australia in 2021, there were 2115 stillbirths representing an overall rate of seven stillbirths per 1000 live births, with six stillbirths occurring every day [2]. The most common cause of death among these stillbirths was congenital anomaly, unexplained antepartum fetal death, maternal conditions, and placental dysfunction [3]. It is important to understand the causes of stillbirth, for families to understand why their baby died, to inform clinical care in subsequent pregnancies, and to develop prevention strategies to decrease the risk and rates of stillbirths [4]. In 2004, the Perinatal Society of Australia and New Zealand (PSANZ) released the first edition of Clinical Practice Guideline (CPG) for perinatal mortality, to enhance a systematic approach to care across Australia and New Zealand including investigations and classification of stillbirths [5]. Subsequent editions continue to align best practice with clinical research, the latest in 2024 [6]. Studies have examined the clinical utility of stillbirth investigations and were limited by the presence of a single review versus a preferred multi‐disciplinary panel [7, 8]. For example, Page et al. described some investigations as pertinent positive, confirming a cause of death or pertinent negative, excluding a cause of death [7]. Excluding suspected cause of death can be as important as confirming a diagnosis as it eliminates potential causes [4]. Building on this prior work, this study sought to systematically assess the clinical utility of stillbirth investigations using a structured and validated approach, in using the Stillbirth Investigation Utility Tool (SIUT) [9].

## 2 Methods

### 2.1 Study Design

A multi‐centre, prospective cohort study across 18 hospitals providing maternity services across Australia (2013–2018) and previously published [10]. Stillbirths at ≥ 20 weeks' gestation and/or ≥ 400 g birthweight were eligible for inclusion. Terminations of pregnancy were excluded. The resulting study population includes 695 stillbirths.

### 2.2 Outcome Measures

Clinical utility of the investigations defined as the proportion of cases in which each test contributed to identification of the COD, either by confirming or excluding.

Change in COD, from laboratory and clinical investigations, to after the placenta and autopsy results were evaluated.

### 2.3 Case Review Procedure

A multi‐disciplinary group of 15 assessors (6 clinical and 9 pathology) reviewed cases and included pathologists, pathology registrars, maternal fetal medicine specialists, neonatologist, an obstetrician, obstetrics & gynaecology registrars, a public health registrar, and a medical officer. The assessors were trained on the use of the SIUT as previously described (Appendix A) [9].

From the group of assessors, each stillbirth was assessed independently by two assessors (one clinical and one pathology) blinded to the COD previously assigned by the participating hospitals. The assessors assessed the clinical utility of each investigation and assigned a COD. Discrepancies between assessors were referred to a tertiary panel comprising an obstetrician, pathologist, and a clinical research midwife for determination of COD.

Each investigation was assigned to one of five categories. The term “useful” was defined as being practically applicable to confirm or exclude COD. For example, maternal full blood count can identify a high white cell count, which can corroborate ascending amniotic infection as a COD. When an investigation was performed it was categorised as ‘useful‐confirmed COD’, ‘useful‐excluded COD’ and ‘not useful’. When an investigation was not performed it was categorised as ‘not performed‐should have been performed’; or ‘not performed‐not necessary’. For tests that confirmed a COD it was found to be a pertinent positive result and for tests that excluded a COD, it was a pertinent negative result. A similar approach was used previously by Korteweg et al. and Page et al. [7, 11].

A stepwise approach was used assigning COD across three groups of tests: Group 1—clinical and laboratory investigations; Group 2—placental pathology; and Group 3—autopsy examination [8]. At the end of each group, the main classification for COD was recorded according to the Perinatal Society of Australia and New Zealand‐Perinatal Death Classification 2018 (PSANZ‐PDC 2018) [5, 12]. A similar approach was used by Miller et al. [8].

### 2.4 Data Collection and Analysis

Panel members entered data into a purpose‐built database [13]. Data analysis was undertaken using R Statistical computing software [14]. Descriptive statistics of the clinical utility of the investigation was performed.

Subgroup analysis was performed according to gestation, presenting clinical scenario and maceration status. Gestation was grouped into five categories: 20.0–23.6, 24.0–27.6, 28.0–31.6, 32.0–36.6 and greater than 37.0. Clinical scenarios were grouped into six categories; unknown; obstetric conditions; intrapartum; hypertensive disorders; fetal growth restriction (FGR) or small for gestational age (SGA) and fetal anomalies. Obstetric conditions included premature rupture of membranes, chorioamnionitis and placental abruption. A similar approach to grouped clinical scenarios was used by Page et al. [7]. Maceration status was defined by the reporting hospitals description of the stillbirth.

### 2.5 Details of Ethics Approval

This study was approved by Queensland Health/Royal Brisbane & Women's Hospital (Reference No.: HREC/12/QRBW/284) and each service.

## 3 Results

### 3.1 Clinical Utility of Investigations for Stillbirth

The frequency of the investigation performed ranged from 0% to 100%, with no case having all investigations performed (Table 1). At least four investigations were performed in every case. Twenty‐one investigations were performed in more than 50% of cases. Four investigations were not performed in any case and the assessors determined the investigations to be unnecessary (needle biopsy, laparoscopy and postmortem ultrasound following birth).

**TABLE 1:** List and proportion of investigations performed according to group.

Group	Investigation	*n* (%) test performed
Group 1 – clinical and laboratory investigations	Comprehensive maternal medical and pregnancy history	626 (90)
	Ultrasound scan for fetal abnormalities	362 (52)
	Ultrasound scan for amniotic fluid assessment	254 (37)
	Amniocentesis for infection	97 (14)
	Amniocentesis for cytogenetics	131 (19)
	Low vaginal/peri‐anal swab	343 (49)
	Full blood count (Hb, WCC, Platelets)	658 (95)
	Blood group & antibody screen	647 (93)
	Feto‐Maternal haemorrhage	502 (72)
	Renal function tests (creatinine, urea)	527 (76)
	Urate	404 (58)
	Liver function tests	544 (78)
	HbA1c	439 (63)
	Thyroid function test	424 (61)
	Bile acids (fasting and/or non‐fasting)	311 (45)
	Cytomegalovirus (CMV)	496 (71)
	Toxoplasma	494 (71)
	Parvovirus B19	452 (65)
	Rubella	549 (79)
	Syphilis	526 (76)
	Anticardiolipin antibodies	453 (65)
	Lupus anticoagulant	441 (63)
	Anti‐Protein C (APC) resistance	386 (56)
	Thrombophilia testing at follow‐up visit	93 (13)
	Fasting homocysteine	54 (8)
	Protein C deficiency	83 (12)
	Protein S deficiency	86 (12)
	Anti‐thrombin III	71 (10)
	Prothrombin G20210A mutation	75 (11)
	Factor V Leiden mutation	82 (12)
	MTHFR 3 mutation	57 (8)
	Clinical photographs taken	239 (34)
	Swabs of ear & throat	324 (47)
	Babygram	251 (36)
	Full blood count with smear	61 (9)
	Chromosomal analysis from the baby—tissue or blood (taken by clinician)	137 (20)
	Newborn Screening Test	45 (6)
	Placental swabs for microbiology by clinician	459 (66)
	Biopsy for cytogenetics taken by clinician	227 (33)
Group 2 – placental pathology	Placental histopathology	656 (94)
	Placental swab for culture taken by pathologist	467 (67)
	Other site culture taken by pathologist	195 (28)
	Tissue for chromosomal analysis taken by pathologist	311 (45)
	External examination by expert in addition to clinician at the birth	139 (20)
	Clinician macroscopic examination of placenta and cord	228 (33)
Group 3 – autopsy examination	Magnetic resonance imaging (MRI)	40 (6)
	Needle biopsy	0
	Laparoscopic	0
	Postmortem ultrasound scan (following birth)	0
	Autopsy—full	268 (39)
	Autopsy—partial	113 (16)

Placental pathology had the highest clinical utility (88%), followed by comprehensive maternal (medical, social, family and pregnancy) history (82%), genetic analysis (75%), maternal blood investigations for infection (including cytomegalovirus (CMV), Toxoplasma, Parvovirus B19, Rubella, Syphilis and full blood count) (57%), fetal‐maternal haemorrhage (FMH) (57%) and full or partial fetal autopsy (47%) (Table 1). The least useful investigations were blood group and antibody screen (64%), liver function tests (41%), renal function tests (40%), and thyroid function tests (36%).

Babygram was performed in 251 cases and considered useful (79%; 198/251). Babygram was performed in the clinical scenario of unknown (47%; 118/251), obstetric conditions (31%; 77/251) and in early stillbirth 20.0–27.6 weeks (51%; 127/251) (Table 2). Magnetic Resonance Imaging (MRI) was performed in 40 cases and highly useful (98%; 39/40). MRI was performed in the clinical scenario of unknown (45%; 18/40), obstetric condition (23%; 9/40), in fetal anomalies (18%; 7/40) and in gestations above 32 weeks (53%; 21/40).

**TABLE 2:** Useful diagnostic test by clinical scenario (695 stillbirths).

	Total (N = # of tests performed)			Unknown (*N* = 300)			Obstetric condition* (*N* = 233)			Intrapartum (*N* = 10)			Hypertensive disorders (*N* = 27)			FGR or SGA (*N* = 53)			Fetal anomalies (*N* = 72)		
	PP *n*	PN *n*	TOTAL *n*, % (95% CI)	PP *n*	PN *n*	TOTAL *n*, % (95% CI)	PP *n*	PN *n*	TOTAL *n*, % (95% CI)	PP *n*	PN *n*	TOTAL *n*, % (95% CI)	PP *n*	PN *n*	TOTAL *n*, % (95% CI)	PP *n*	PN *n*	TOTAL *n*, % (95% CI)	PP *n*	PN *n*	TOTAL n, % (95% CI)
	% (95% CI)	% (95% CI)		% (95% CI)	% (95% CI)		% (95% CI)	% (95% CI)		% (95% CI)	% (95% CI)		% (95% CI)	% (95% CI)		% (95% CI)	% (95% CI)		% (95% CI)	% (95% CI)	
Comprehensive maternal history	325,46.8 (43.1–50.6)	242, 34.8 (31.3–38.5)	567, 81.6 (78.5–84.4)	54, 18.0 (13.8–22.9)	178, 59.3 (53.7–65.1)	232, 77.3 (72.2–82.0)	155, 66.5 (60.1–72.25)	82, 35.2 (29.1–41.7)	197, 84.5 (79.3–88.9)	1, 10 (25–44.5)	6, 60 (26.4–87.8)	7, 70 (34.8–93.3)	20, 74.1 (53.7–88.9)	4,14.8 (4.2–33.7)	24, 88.9 (70.8–97.6)	19, 35.8 (23.1–50.2)	25, 47.2 (33.3–61.4)	44, 83.0 (70.2–91.9)	28, 38.9 (27.6–51.1)	33, 45.8 (34.0–58.0)	61, 84.7 (74.3–92.1)
Feto‐maternal haemorrhage^a^	30, 4.3 (2.9–6.1)	368, 52.9 (49.2–56.7)	398, 57.3 (53.5–61.0)	12, 4.0 (2.1–6.9)	188, 62.7 (56.9–68.2)	200, 66.7 (61.0–72.0)	2, 0.8 (0.1–3.1)	82, 35.2 (29.1–41.7)	84, 36.3 (29.9–42.6)	1, 10 (25–44.5)	5, 50 (18.7–81.3)	6, 60 (26.4–87.8)	0, 0 (0.0–12.8)	13, 48.2 (28.7–68.1)	13, 48.2 (28.7–68.1)	0, 0 (0.0–6.7)	29, 54.7 (40.4–68.4)	29, 54.7 (40.4–68.4)	2, 2.7 (0.3–9.7)	31, 43.4 (31.4–55.3)	33, 45.8 (34.0–58.0)
Maternal bloods for infection^b^	39, 5.6 (4.0–7.6)	360, 51.8 (48.0–55.6)	399, 57.4 (53.6–61.1)	0, 0.0 (0.0–1.2)	197, 65.7 (60.0–71.0)	197, 65.7 (60.0–71.0)	3, 1.9 (0.2–3.7)	133, 57.1 (50.5–63.5)	136, 58.4 (51.8–64.8)	1, 10 (25–44.5)	8, 80 (44.4–97.5)	9, 90 (55.5–99.7)	4,14.8 (4.2–33.7)	15, 55.6 (35.3–74.5)	15, 55.6 (35.3–74.5)	0, 0 (0.0–6.7)	35, 66.0 (51.7–78.5)	35, 66.0 (51.7–78.5)	0, 0 (0.0–5.0)	34, 47.3 (35.3–59.3)	34, 47.3 (35.3–59.3)
Babygram	20, 2.9 (1.8–4.4)	178, 25.6 (22.4–29.0)	198, 28.5 (25.2–32.0)	7, 2.3 (0.9–4.8)	85, 28.3 (23.3–33.8)	92, 30.7 (25.5–36.2)	3, 1.9 (0.2–3.7)	59, 25.3 (19.9–31.4)	62, 26.6 (21.1–32.8)	0 (0.0–30.8)	1, 10 (25–44.5)	3, 30 (6.7–65.2)	0, 0 (0.0–12.8)	4,14.8 (4.2–33.7)	4,14.8 (4.2–33.7)	1, 1.9 (0.0–10.1)	16, 30.2 (18.3–44.3)	17, 32.1 (20.0–46.3)	9, 12.5 (5.9–22.4)	11, 15.3 (7.9–25.9)	20, 27.8 (17.9–39.6)
Genetic analysis^c^	45, 6.5 (4.8–8.6)	478, 68.8 (65.2–72.2)	523, 75.3 (72.0–78.4)	17, 5.7 (3.3–8.9)	241, 80.3 (75.4–84.7)	258, 86 (82.2–90.2)	10, 4.3 (2.1–7.8)	140, 60 (53.5–66.4)	150, 64.4 (57.9–70.5)	0 (0.0–30.8)	6, 60 (26.4–87.8)	6, 60 (26.4–87.8)	0, 0 (0.0–12.8)	20, 74.1 (53.7–88.9)	20, 74.1 (53.7–88.9)	3, 5.7 (1.2–15.7)	41, 77.4 (63.8–87.7)	44, 83.0 (70.2–91.9)	15, 20.8 (12.2–32.0)	31, 43.4 (31.4–55.3)	46, 63.9 (51.7–75.9)
Placental histopathology	379, 54.5 (50.7–58.3)	232, 33.4 (29.9–37.0)	611, 87.9 (85.3–90.2)	128, 42.8 (37.1–48.6)	131, 43.8 (38.‐49.6)	259, 86.0 (81.6–89.7)	166, 71.2 (65.0–77.0)	47, 20.2 (15.2–26.0)	213, 91.4 (87.1–94.7)	7, 70 (34.8–93.3)	3, 30 (6.7–65.2)	10, 100 (69.2–100)	19, 70.4 (49.8–86.2)	5, 18.5 (6.3–38.1)	24, 88.9 (70.8–97.6)	35, 66.0 (51.7–78.5)	14, 26.4 (15.3–40.3)	49, 92.5 (81.8–97.9)	24, 33.3 (22.7–45.4)	32, 44.4 (32.7–56.6)	56, 77.8 (66.4–86.7)
MRI	16, 2.3 (1.3–3.7)	23, 3.3 (2.1–4.9)	39, 5.6 (4.0–7.6)	5, 1.7 (0.5–3.8)	12, 4 (2.1–6.9)	17, 5.7 (3.3–8.9)	3, 1.9 (0.2–3.7)	6, 2.6 (0.9–5.5)	9, 3.9 (1.8–7.2)	0 (0.0–30.8)	1, 10 (25–44.5)	1, 10 (25–44.5)	0, 0 (0.0–12.8)	3, 11.1 (2.4–29.2)	3, 11.1 (2.4–29.2)	1, 1.9 (0.0–10.1)	0, 0 (0.0–6.7)	1, 1.9 (0.0–10.1)	7, 9.7 (4.0–19.0)	0, 0 (0.0–5.0)	7, 9.7 (4.0–19.0)
Autopsy—full	136, 19.6 (16.7–22.7)	120, 17.3 (14.5–20.3)	256, 36.8 (33.2–40.5)	43, 14.3 (10.6–18.9)	86, 28.7 (23.6–34.1)	129, 43.0 (37.3–48.8)	41, 17.6 (12.9–23.1)	30, 12.9 (8.9–17.9)	71, 30.5 (24.6–36.8)	1, 10 (25–44.5)	2, 20 (2.5–55.6)	3, 30 (6.7–65.2)	6, 22.2 (8.6–42.3)	2, 7.4 (0.9–24.3)	8, 29.6 (13.8–50.2)	12, 22.6 (12.3–36.2)	10, 18.9 (9.4–32.0)	22, 41.5 (28.1–55.9)	18, 25 (15.5–36.6)	5, 6.9 (2.3–15.5)	23, 31.9 (21.4–44.0)
Autopsy—partial	22, 3.2 (2.0–4.8)	49, 7.1 (5.3–9.2)	71, 10.2 (8.1–12.7)	6, 2.0 (0.7–4.3)	32, 10.7 (7.4–14.7)	38, 12.7 (9.1–17.0)	10, 4.3 (2.1–7.8)	10, 4.3 (2.1–7.8)	20, 8.6 (5.3–12.9)	0 (0.0–30.8)	1, 10 (25–44.5)	1, 10 (25–44.5)	1, 3.7 (0.0–19.0)	1, 3.7 (0.0–19.0)	2, 7.4 (0.9–24.3)	1, 1.9 (0.0–10.1)	3, 5.7 (1.2–15.7)	4, 7.5 (2.1–18.2)	5, 6.9 (2.3–15.5)	1,1.4 (0.0–7.5)	6, 8.3 (3.1–17.3)

Abbreviations: CI, confidence interval; FGR, fetal growth restriction; PN, pertinent negative result; PP, pertinent positive result; SGA, small for gestational age.

* Obstetric conditions include preterm labour, premature preterm rupture of membranes, chorioamnionitis.

^a^ Denominator is the number of stillbirths in each clinical scenario. Feto‐Maternal haemorrhage ‐Kleihauer‐Betke or flow cytometry.

^b^ Maternal bloods for infection including following organisms CMV, Toxoplasma, Parvovirus B19, Rubella, Syphilis and full blood count.

^c^ Genetic analysis—Microarray, Karyotype or both.

The clinical utility of investigations was stratified by presenting clinical scenario (Table 2). For cases with an unknown clinical scenario, the highest clinical utility investigations were placental pathology (86%; 259/300) and genetic analysis (86%; 258/300). For obstetric conditions, placental pathology (91%; 213/233), genetic analysis (64%; 150/233) and maternal bloods for infection (58%; 136/233) were most useful. In an intrapartum death, the highest clinical utility investigations were placental pathology (100%; 10/10), maternal bloods for infection (90%; 9/10) and FMH (60%; 6/10). For cases presenting with hypertensive disorders, placental pathology (89%; 24/27) and genetic analysis (74%; 20/27) were the most useful. Autopsy examination had the lowest clinical utility at 37% (10/27) for this scenario compared to other clinical scenarios. For clinical FGR or SGA, placental pathology (92%; 49/53) and genetic analysis (91%; 48/53) had the highest utility. For babies with suspected fetal anomaly on antenatal ultrasound or prenatal genetic screening, the most useful investigations were placental pathology 78% (56/72), genetic analysis 64% (46/72) and maternal bloods for infection 47% (34/72).

The clinical utility of placental pathology, genetic analysis, and autopsy were stratified by maceration status. There was no clear deterioration in the utility of tests with increasing maceration. Autopsy had high clinical utility in cases of severe maceration 68% (56/82) and was slightly higher in excluding COD compared to confirming COD. Minimal difference was observed in placental pathology ranging from 90% to 93%, with the placenta being a pertinent positive. Genetic analysis had the highest clinical utility in cases with moderate maceration 97% (125/129), reducing slightly to 95% (78/82) in severe maceration, excluding the COD.

**FIGURE 1:**
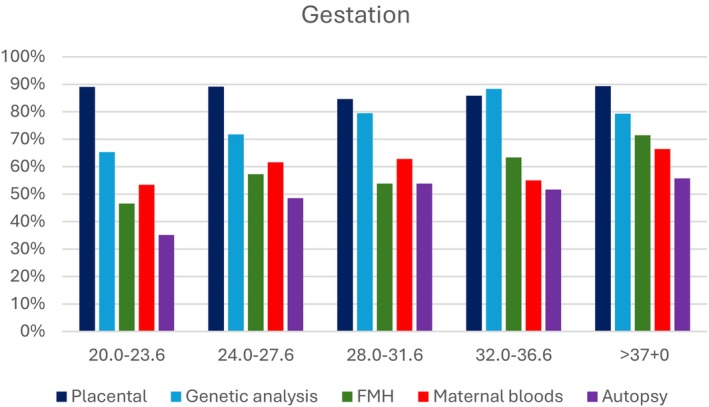
Clinical utility of placental pathology, genetic analysis, feto‐maternal haemorrhage (FMH), maternal bloods for infection and autopsy investigations stratified by gestation.

The clinical utility of investigations was stratified by gestation (Figure 1). Placental pathology showed little variation in usefulness between gestations with a range of 86%–89%. Autopsy examination became more useful, increasing from 35% to 56%, as gestation progressed, similar to FMH, which became more useful as gestation progressed from 47% to 71%. Genetic analysis was most useful from 32.0–36.6 weeks at 88% and least useful from 20.0 to 23.6 weeks at 65%.

**FIGURE 2:**
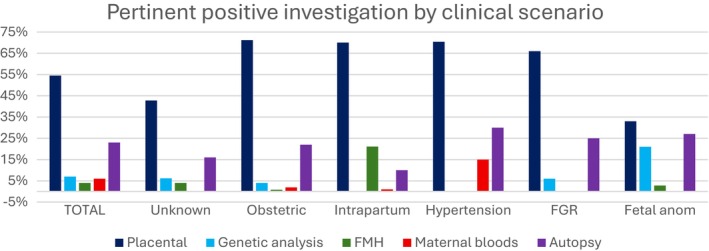
Pertinent positive (confirming cause of death) for placental pathology, genetic analysis, FMH, maternal bloods for infection and autopsy investigations stratified by presenting clinical scenario.

When the utility was reviewed for pertinent positive results only, placental pathology and autopsy had the highest utility in all clinical scenarios except for the intrapartum scenario (Figure 2) and in all gestations.

### 3.2 Change in Cause of Death

The COD changed after group 2 investigations (placenta report) were reviewed in 310 cases (47%) with a COD specific to the placenta identified in 301/310 (97%) and infection in 9/310 (3%). Autopsy examination was performed in 322 cases (46%) and found to be useful in 287 cases (89%). The autopsy was found to be not useful when the COD was identified by other testing or when no COD was determined. Despite full investigations, 167 (24%) cases were found to be unexplained (PSANZ PDC 2018 11.1).

## 4 Discussion

### 4.1 Main Findings

This is the largest Australian study to assess the clinical utility of investigations in stillbirths. The most useful investigations found to establish or exclude a COD were placental pathology, comprehensive maternal history, genetic analysis, maternal blood investigations for infection, assessment for fetal‐maternal haemorrhage and fetal autopsy.

These findings are consistent with two other large studies in The Netherlands and The United States (U.S.). Despite differing classification of death systems across all settings, similar variation in rates of full autopsy and other diagnostic tests was observed [7, 11]. In The Netherlands, Korteweg et al. studied 1025 fetal deaths from 50 hospitals and found the most valuable investigation in establishing or excluding a COD according to the Tulip classification system to be placental pathology (95.7%), fetal autopsy (72.6%) and genetic analysis (29%) [11]. In this study, 68% of stillbirths underwent either a full or partial autopsy. In the U.S. Page et al. assessed 512 stillbirths and measured value by the usefulness of the investigation in establishing a COD using the Initial Causes of Fetal Death instrument (INCODE). They found the most valuable investigations were placental pathology (64.6%), full autopsy examination (42.4%) and genetic analysis (11.9%) with the utility of each test varying by clinical scenario [7]. Most tests were pertinent positive results, with FMH and antiphospholipid antibodies testing being pertinent negative results. This contrasts with our study that found most investigations to be pertinent negative results, and our pertinent positive results were slightly lower. Our study had a similar high utility of placental pathology as in The Netherlands cohort but lower utility of the fetal autopsy, the later more in keeping with the findings of the U.S. cohort.

The American cohort is the only previous study to review clinical utility by presenting clinical scenario. They found placenta pathology and fetal autopsy to be useful in all clinical scenarios. Genetic analysis useful in FGR, obstetric, unknown clinical scenario and FMH was useful in intrapartum clinical scenarios. Our study had similar findings for utility by clinical scenario.

Placental pathology was pertinent positive in all clinical scenarios except fetal anomalies. Fetal autopsy was pertinent positive in cases with hypertensive disorders, fetal growth restriction, obstetric conditions, and fetal anomalies. It was pertinent negative in an unknown clinical scenario and intrapartum deaths. Even though it excluded a cause of death, fetal autopsy can provide important information and can provide comfort and reassurance to grieving families as well as guidance for planning subsequent pregnancies. Genetic testing was pertinent negative in all clinical scenarios. FMH was pertinent negative in all clinical scenarios; however, it was most pertinent positive in the intrapartum clinical scenario.

Our findings also corroborate previous studies that identified the significance of placental pathology in identifying CODs in stillbirths [7, 8, 11, 15, 16]. Miller et al. found placental pathology identified a probable COD in 88/144 (61%) of cases and changed case management in 52/144 (36%). The INCODE classification system, used in the studies by Miller et al. and Page et al. has separate categories for placenta with or without fetal infection and pathologic placental conditions [7, 8, 17]. Darouich et al. similarly found a COD attributed to the placenta in 89/147 (61%) of stillbirths using the Tulip classification system [15, 18]. This is further supported by a 2021 study from Cullen et al. who examined the placenta first, and identified a COD in the placenta and cord in 17/25 (68%) of cases [16].

Our results support the recommendations of a selective approach to stillbirth investigations [19]. A selective approach provides an individualised approach to each stillbirth, based on the presenting clinical scenario of the mother and/or baby, on the investigations that will provide the most value to determining the cause of death. An Australian study found an average of $4200 was spent on investigations for each stillbirth, with the most expensive investigations being autopsy and genetic analysis [20]. A selective approach has the potential to reduce the average cost of investigating stillbirths, minimise the economic impact of unnecessary investigation and reduce the turnaround times of results.

### 4.2 Strengths and Limitations

This is the largest Australian study to examine the clinical utility of stillbirth investigations using a panel with a range of clinical experience from a consultant to a junior medical officer. The size of the cohort makes it the second largest study worldwide and the largest to review utility of stillbirth investigations by presenting the clinical scenario.

The study was undertaken during 2013–2018, with the recommended guidelines for investigation being very different from the current guidelines. This introduces bias into our findings as the current guidelines use a selective/sequential investigation protocol as opposed to all investigations that were relevant during the study time. The findings of this study support current PSANZ guidelines in having a selective approach to stillbirth investigations.

Whilst the study was undertaken during 2013–2018, we reviewed the COD according to the latest PSANZ‐PDC 2018, rather than the PSANZ‐PDC 2009, that was relevant at the time. This introduces bias as the current version has a separate category for placental dysfunction or causative placental pathology [12].

There are many investigations where no data were uploaded, and it was assumed the test was not performed. Future studies should consider exclusion criteria when assessing investigations to provide the most accurate utility assessment. Further, each investigation was performed according to local procedures, which varied systematically for all participating hospital pathology laboratories. These factors could limit the overall clinical utility of the investigation.

We found placental pathology to be one of the most important clinical investigations and recommend it be performed for all cases of stillbirths. Genetic analysis is the second most important clinical investigation. Importantly, genetic analysis can be performed on the placenta successfully [21]. Our study found when an autopsy is performed, it is useful in 89% of cases. Even if no COD is found, the negative results of an autopsy provide important information and can provide comfort and reassurance to grieving families as well as guidance for planning subsequent pregnancies. Placental pathology and genetic analysis are useful in all clinical scenarios. Maternal bloods for infection are useful in obstetric, intrapartum and fetal anomalies. FMH is useful in Intrapartum clinical scenario. Fetal autopsy is most useful in unknown clinical scenarios, in particular term stillbirths. This study supports the recommendation for core investigations, comprising a comprehensive medical history and examination (including blood tests), placental pathology and genetic analysis (if not performed during pregnancy), as the most useful for investigating stillbirths. Further specific investigations, based on the presenting clinical scenario and findings from the core investigations, can guide a more individualised and selective approach to each stillbirth.

## Funding

This study is supported under NHMRC Project Grant #1029613. “Investigating Causes of Stillbirths: A Prospective Cohort Study Examining Use and Effectiveness of a Comprehensive Investigation.” This study is supported by the NHMRC Stillbirth Centre of Research Excellence (AP1116640). V.F. is supported by NHMRC Investigator Grants (APP2010136). Support by Mater Foundation is kindly acknowledged.

## Ethics Statement

This study was approved by the Mater Health Services Human Research Ethics Committee on 20 December 2011 (Reference No.: HREC/1745 M), Queensland Health/Royal Brisbane & Women's Hospital on 17 December 2012 (Reference No.: HREC/12/QRBW/284), ACT Health HREC on 5 November 2012 (Reference No.: ETH.10.12.220), Northern Sydney Local Health District HREC on 31 January 2013 (Ref No. 1212‐411 M), HREC of Northern Territory Department of Health and Menzies School of Health Research (Reference No.: HOMER‐2012‐1876), Aboriginal Health Research Ethics Committee (AHREC) of South Australia on 5 November 2012 (Reference No.: 04–12‐480), Women's & Children's Hospital Network (WCHN) HREC on 5 December 2012 (Reference No.: HREC/12/WCHN/69), University of Tasmania HREC Tasmania Network on 30 November 2012 (Reference No.: H0012864), Mercy Health HREC (Victoria) on 11 June 2013 (Reference No.: R13/07), and Western Australia Aboriginal Health Ethics Committee on 19 November 2012 (Reference No.: 447). Due to delays and complications during the HREC review process, no stillbirths were recruited from health facilities in Western Australia.

## Conflicts of Interest

The authors declare no conflicts of interest.

## Data Availability

The data that support the findings of this study are available from the corresponding author upon reasonable request.

## Appendix A

**Stillbirth Investigation Utility Tool**

**Table:**

Investigation number	Investigation	Useful		Not Useful	Not performed	
		Confirmed COD	Ruled out COD		Should have been performed	Not necessary
Phase 1						
Maternal investigations						
1	Comprehensive maternal medical and pregnancy history	☐	☐	☐	☐	☐
2	Ultrasound scan for fetal abnormalities	☐	☐	☐	☐	☐
3	Ultrasound scan for amniotic fluid assessment	☐	☐	☐	☐	☐
4	Amniocentesis for infection	☐	☐	☐	☐	☐
5	Amniocentesis for cytogenetics	☐	☐	☐	☐	☐
6	Low vaginal/peri‐anal swab	☐	☐	☐	☐	☐
7	Full blood count (Hb, WCC, Platelets)	☐	☐	☐	☐	☐
8	Blood group & antibody screen	☐	☐	☐	☐	☐
9	Maternal‐Fetal haemorrhage	☐	☐	☐	☐	☐
10	Renal function tests (creatinine, urea)	☐	☐	☐	☐	☐
11	Urate	☐	☐	☐	☐	☐
12	Liver function tests	☐	☐	☐	☐	☐
13	HbA1c	☐	☐	☐	☐	☐
14	Thyroid function test	☐	☐	☐	☐	☐
15	Bile acids (fasting and/or non‐fasting)	☐	☐	☐	☐	☐
16	CMV	☐	☐	☐	☐	☐
17	Toxoplasma	☐	☐	☐	☐	☐
18	Parvovirus B19	☐	☐	☐	☐	☐
19	Rubella	☐	☐	☐	☐	☐
20	Syphilis	☐	☐	☐	☐	☐
21	Anticardiolipin antibodies	☐	☐	☐	☐	☐
22	Lupus anticoagulant	☐	☐	☐	☐	☐
23	APC resistance	☐	☐	☐	☐	☐
24	Other maternal investigations performed	☐	☐	☐	☐	☐
25	Thrombophilia testing at follow‐up visit	☐	☐	☐	☐	☐
26	Fasting homocysteine	☐	☐	☐	☐	☐
27	Protein C deficiency	☐	☐	☐	☐	☐
28	Protein S deficiency	☐	☐	☐	☐	☐
29	Anti‐thrombin III	☐	☐	☐	☐	☐
30	Prothrombin G20210A mutation	☐	☐	☐	☐	☐
31	Factor V Leiden mutation	☐	☐	☐	☐	☐
32	MTHFR 3 mutation	☐	☐	☐	☐	☐
Baby investigations						
33	Clinical photographs taken	☐	☐	☐	☐	☐
34	Swabs of ear & throat	☐	☐	☐	☐	☐
35	Babygram	☐	☐	☐	☐	☐
36	Full blood count with smear	☐	☐	☐	☐	☐
37	Chromosomal analysis from the baby—tissue or blood (taken by clinician)	☐	☐	☐	☐	☐
38	Newborn Screening Test	☐	☐	☐	☐	☐
39	Placental swabs for microbiology by clinician	☐	☐	☐	☐	☐
40	Biopsy for cytogenetics taken by clinician	☐	☐	☐	☐	☐
Classification of COD						
Phase 2 – Placenta report provided						
41	Placental histopathology	☐	☐	☐	☐	☐
42	Placental swab for culture taken by pathologist	☐	☐	☐	☐	☐
43	Other site culture taken by pathologist	☐	☐	☐	☐	☐
44	Tissue for chromosomal analysis taken by pathologist	☐	☐	☐	☐	☐
Classification of COD						
Phase 3 – Autopsy report provided						
45	MRI	☐	☐	☐	☐	☐
46	Needle biopsy	☐	☐	☐	☐	☐
47	Laparoscopic	☐	☐	☐	☐	☐
48	Other technique	☐	☐	☐	☐	☐
49	Postmortem ultrasound scan (following birth)	☐	☐	☐	☐	☐
50	External examination by expert in addition to clinician at the birth	☐	☐	☐	☐	☐
51	Clinician macroscopic examination of placenta and cord	☐	☐	☐	☐	☐
52	Autopsy—full	☐	☐	☐	☐	☐
53	Autopsy—partial	☐	☐	☐	☐	☐
Classification of COD						
